# Early locomotor activity in broilers and the relationship with body weight gain

**DOI:** 10.1016/j.psj.2022.102086

**Published:** 2022-07-30

**Authors:** Malou van der Sluis, Lucy Asher, T. Bas Rodenburg, Yvette de Haas, Britt de Klerk, Esther D. Ellen

**Affiliations:** ⁎ Animal Breeding and Genomics, Wageningen University & Research, 6700 AH Wageningen, The Netherlands; † Animals in Science and Society, Faculty of Veterinary Medicine, Utrecht University, 3508 TD Utrecht, The Netherlands; ‡ School of Natural and Environmental Sciences, Newcastle University, NE1 7RU, Newcastle upon Tyne, United Kingdom; § Adaptation Physiology Group, Wageningen University & Research, 6700 AH Wageningen, The Netherlands; # Cobb Europe, 5831 GH Boxmeer, The Netherlands

**Keywords:** broiler, activity, body weight, tracking, entropy

## Abstract

Fast-growing broilers are relatively inactive and this is thought to be a result of selection for high growth rates. This reduced activity level is considered a major cause of leg weakness and associated leg health problems. Increased activity, especially early in life, is suggested to have positive effects on leg health, but the relationship between early activity and growth is unclear. A clearer understanding of the relationship between activity early in life and body weight gain could help determine how selecting on increased early activity could affect body weight gain in broilers. Here, a radio frequency identification (**RFID**) tracking system was implemented to record daily individual broiler activity throughout life, in 5 production rounds. As mean activity levels alone do not capture the variation in activity over time, multiple (dynamic) descriptors of activity were determined based on the individual birds’ daily distances moved, focusing on the period from 0 to 15 days old. The mean, skewness, root mean square error (**RMSE**), autocorrelation, and entropy of (deviations in) activity were determined at the individual level, as well as the average daily gain (**ADG**). Relationships between activity descriptors and ADG were determined for 318 birds. Both when combining the data from the different production rounds and when taking production round and start weight into account, a negative relationship between ADG and RMSE was observed, indicating that birds that were more variable in their activity levels had a lower ADG. However, the activity descriptors, in combination with recording round and start weight, explained only a small part (8%) of the variation in ADG. Therefore, it is recommended for future research to also record other factors affecting ADG (e.g., type of feed provided and feed intake) and to model growth curves. Overall, this study suggests that increasing early activity does not necessarily negatively affect body weight gain. This could contribute to improved broiler health and welfare if selecting for increased activity has the expected positive effects on leg health.

## INTRODUCTION

The growth rate of broilers has changed considerably over the years, as a consequence of selection for an increased growth rate and feed conversion, as well as changes in the diet. Havenstein et al. (2003) compared broiler strains from 1957 and 2001 on diets representative of those times, and estimated that the 2001 strain on the 2001 diet would reach a body weight of 1,815 grams at 32 d of age, while the 1957 strain on the 1957 diet would only have reached this same body weight at 101 d of age. The selection for high body weight gain is suggested to have resulted in a reduced activity level (Bizeray et al., 2000; Weeks et al., 2000). Dixon (2020) compared 3 faster growing broiler breeds to a slower growing breed, at the same age, and observed that the faster growing breeds spent more time sitting and less time in locomotion later in life, from 23 days old onwards for sitting and from 30 days old onwards for locomotion. Similar observations were reported by Bokkers and Koene (2003), indicating that fast-growing broilers are relatively inactive.

Not only compared to slow-growing broilers, but also within fast-growing broilers, differences in activity have been observed that have been hypothesized to be related to body weight. For example, it has been observed that birds categorized as lightweight at approximately 2-wk-old showed a higher mean activity level over the period from 16 to 32 days old, than birds categorized as heavyweight (van der Sluis et al., 2019). Bokkers et al. (2007) tested fast-growing broilers in an operant runway test and observed that lightweight birds walked longer distances for a food reward than heavyweight birds. These studies suggest that higher body weights are linked to lower activity levels.

Low activity levels are considered to be one of the main causes of leg weakness (Bessei, 2006), which is a general term to describe multiple pathological states that result in impaired walking ability in broilers (Butterworth, 1999). Leg weakness might be painful for the affected birds (Danbury et al., 2000). Moreover, in severe cases, birds may have difficulties competing with others for resources and may be restricted in performing specific behaviors, like dustbathing (Kestin et al., 1992; Vestergaard and Sanotra, 1999). Increasing locomotor activity levels may help to prevent leg problems (Reiter and Bessei, 2009). Different approaches for increasing activity, and hereby reducing leg problems, have been studied, for example, using different types of environmental enrichment (Vasdal et al., 2019), sequential feeding (Bizeray et al., 2002), or elevated platforms (Kaukonen et al., 2017). It has been suggested that specifically early in life increases in activity can improve leg health (e.g., Bizeray et al. (2000)), as the first weeks after hatching form a critical period in terms of bone development (Sanchez-Rodriguez et al., 2019), and a recent study has shown that activity is heritable in broilers, with an estimated heritability of 0.31 ± 0.11 across the full production period (Ellen et al., *unpublished data*). However, it is unclear how early activity relates to body weight gain.

A clearer understanding of the relationship between activity early in life and body weight later in life would be valuable, as this would help to gain insight into how selecting on increased early activity affects growth, a commercially important trait, in broilers. However, as emphasized in a review by Asher et al. (2009), behavior is complex and multidimensional, and consequently the use of multidisciplinary approaches for behavior analyses is encouraged, and already implemented in studies on chicken behavior (e.g., Rutherford et al., 2003). Mean activity levels alone may provide insufficient insight to detect early differences in activity, and therefore, additional dynamic activity descriptors can have great added value. For example, Dawkins et al. (2012) studied optical flow patterns in broiler flocks, in relation to welfare measurements such as gait. They observed no correlation between the mean gait score and the mean optical flow, but did observe correlations between the mean gait score and the skew and kurtosis of optical flow. This shows that dynamic descriptors of activity can be more sensitive than mean activity levels alone.

In this study, dynamic descriptors of activity were studied in relation to body weight gain. We implemented an earlier-validated radio frequency identification (**RFID**) tracking system to record the activity of individual broilers throughout life (van der Sluis et al., 2020). From these activity recordings, individual activity levels, here calculated as distances moved, were determined for the first 2 wk after hatching. We calculated the mean, skewness, root mean square error (**RMSE**), and autocorrelation of (deviations in) activity, as these descriptors of traits have been suggested to provide indications for resilience (Berghof et al., 2019b; van der Zande et al., 2020) and have potential to be implemented on repeatedly measured traits. For example, several of these indicators were calculated for body weight deviations in layer chickens (Berghof et al. 2019a) and for activity levels in pigs (van der Zande et al., 2020). We furthermore included entropy of activity, to assess the regularity of activity within days. Existing implementations of entropy in animal behavior are limited, but it has for example been observed that spontaneously hypertensive rats displayed a higher complexity of movement time series, that is, higher entropy, than control rats (Fasmer and Johansen, 2016). To examine whether these descriptors are related to broiler growth, we recorded the body weight of the birds every week and calculated the average daily gain (**ADG**) across the full production period. The overall aim of this study was to investigate the relationship between early activity and body weight gain, by determining dynamic descriptors of activity and combining the activity and body weight records. The broilers were kept under commercial conditions and no additional challenges were implemented to achieve contrast between individuals. Therefore, we expected only small effects in terms of activity descriptors. Under the assumption that a lower ADG is indicative of reduced welfare, the expectation was that a lower ADG would be linked to: 1) a lower mean activity; 2) a reduced (due to consistently low activity) or increased (due to some uncharacteristically inactive days) skewness of activity; 3) an increased RMSE due to more deviations in activity levels; 4) an increased autocorrelation of activity deviations due to activity deviations on subsequent days becoming more related; and 5) an increased entropy of activity due to a less regular daily activity pattern.

## MATERIALS AND METHODS

### Ethical Statement

Data were collected on a broiler farm in the Netherlands, under control of Cobb Europe. Cobb Europe complies with the Dutch legislation on animal welfare. This study is considered not to be an animal experiment under the Law on Animal Experiments, as confirmed by the local Animal Welfare Body (20th of June, 2018, Lelystad, the Netherlands).

### Location and Subjects

**Figure 1:**
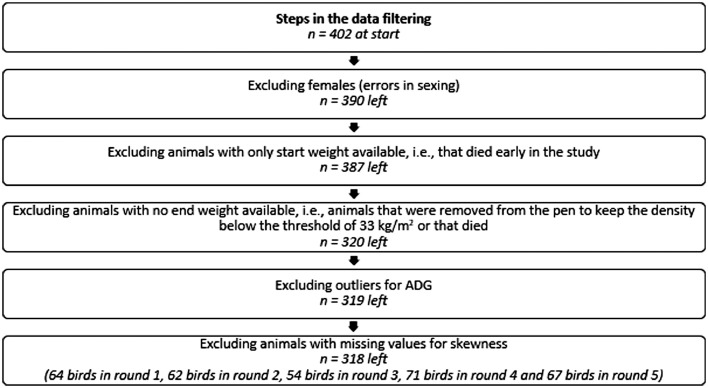
Overview of the steps in the data filtering and resulting sample sizes.

In total, the activity of 402 broiler chickens was tracked on-farm with an RFID system (van der Sluis et al., 2020). These 402 broilers were divided over 5 consecutive production rounds. The initial number of birds and the recording length differed per round and are shown in Table 1. Only male broilers were aimed to be included in this study, and therefore females that were included due to errors in sexing were excluded from the data, as well as birds that died during the study (Figure 1; see also Ellen et al. (*unpublished data*) for a more elaborate description of the data filtering steps).

**Table 1:** Overview of the number of broilers at the start of the round and the age of the birds at the start and end of the round, per recorded production round.

Round	Age at start (days)	Age at end (days)	n_birds_ start
1	1	36	80
2	1	33	82
3	0	35	82
4	1	35	78
5	1	33	80
Total			402

### Housing

In all five production rounds, the broilers were housed in a rectangular pen with a size of approximately 1.8 × 2.6 m (i.e., 4.7 m^2^), which was fitted with a passive RFID system (van der Sluis et al., 2020). During the production period, the weight of the birds was monitored closely. Before the density in the pen would reach approximately 33 kg/m^2^, some birds were removed from the pen and housed elsewhere, to avoid reduced activity due to high densities in the pen, yet at the same time aiming to remain close to this density to mimic commercial conditions as much as possible. Birds that were removed were excluded from the analyses, as no end weight was available for these birds (Figure 1). In the pen, feed and water were provided ad libitum and wood shavings were provided as bedding. Starter, grower and finisher feed types were provided according to the common Cobb broiler feeding scheme (Cobb, 2018). The birds were kept under a commercial lighting and temperature schedule, with dark periods from 23:00 to 03:00 and from 05:00 to 07:00, and were vaccinated according to common practice (Cobb, 2018).

### Body Weight Records

Weighing of the birds was performed weekly, starting on the day of hatching and ending on the last day of the respective round (see Table 1). Weights were determined with 5-gram precision, except for the start weight, which was determined with 2-gram precision in rounds 1 and 3 and with one-gram precision in rounds 2, 4, and 5. The ADG was calculated as:

$$ADG=\;\frac{\left(body\;weight_{end}-body\;weight_{start}\right)}{\left(age\;in\;days_{end}-age\;in\;days_{start}\right)}$$

The age at which the end weight was recorded differed per round due to unequal lengths of rounds. By looking at ADG instead of end weight, a correction for this unequal length of rounds was made. The ADG across the period from 0–1 to 33–36 days old was used as the descriptor of body weight gain in this study. Extreme outliers for ADG were identified using a threshold of four times the standard deviation, and were determined within rounds. The outliers were identified using all animals in that round and ADG outliers were removed row-wise, meaning that the one animal for which this was the case was completely removed from the data (Figure 1). At this point, data for a total of 319 birds were available for analysis.

### Activity Records

To record the activity levels of the broilers, a passive RFID system from Dorset ID (Dorset Identification B.V., Aalten, the Netherlands) was implemented. All birds were fitted with an RFID tag, with a size of approximately 15 × 3.7 mm and a weight of less than one gram. These tags were attached to the birds’ legs using rubber bands and tape, and were changed to a larger size once during the study and checked every couple of days. The broilers’ pen was fitted with 30 high frequency antennas, with a size of 32 by 41 cm each, in a grid on the underside of a false floor. The RFID system could register the presence of RFID tags at these antennas and stored a log file that included a timestamp, the ID code of the tag and the location, that is, antenna, at which the tag was registered. The recording frequency used here was generally one sample per second for all antennas, with exception of the instances in which antenna switches occurred and 2 different registrations within the same second could be observed. The RFID system and its validation, showing among other things a rank correlation between video and RFID of 0.82 in terms of recorded distances moved, are described in more detail in Van der Sluis et al. (2020). In this study, RFID recordings were generally made continuously for 24 h per day, but the periods in which the birds were not all in the pen, for example due to weighing or switching the leg bands of the birds, were excluded. Furthermore, corrections for missing data, for example, due to technical problems, were made at the group-level when no RFID data was recorded for more than five consecutive minutes. Very occasionally, this resulted in a day without data available. Due to technical difficulties in round 1, there were relatively many short periods of missing data in this round. Furthermore, due to wet bedding in round 1, which was avoided in later rounds, the antennas underneath the drinker could not detect the presence of tags well, resulting in lower recorded activity levels in this round. A round effect was taken into account in part of the statistical analyses.

### Activity Calculations

**Figure 2:**
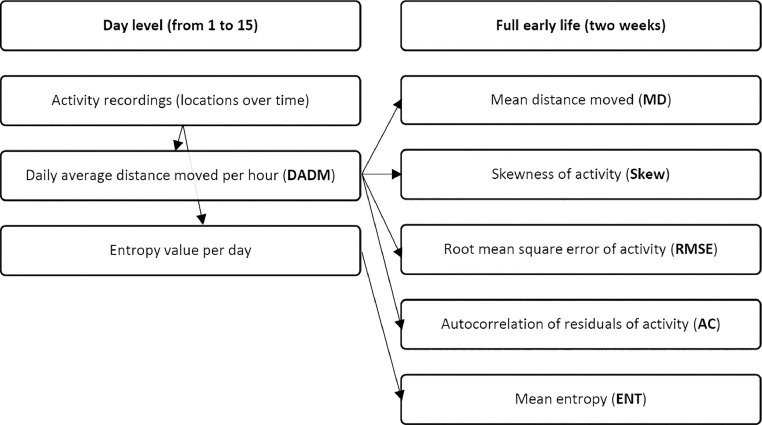
Schematic overview of the calculations of the activity descriptors.

The activity calculations in this study were made using only the data on the main light period from 07.00 to 23.00 each day, since broilers are observed to be mainly active during the light periods and relatively inactive during the dark periods (e.g., Nielsen et al. (2004)) and we observed a strong correlation between the total distances moved across 24 h and across the main light period in the full dataset (tau = 0.953 [95% CI 0.952–0.954], *P* < 0.001). Given the interest in activity early in the production period, only data from the first 2 wk after hatching (up to and including 15 days old) were studied here. From the RFID log, multiple activity indicators were calculated, for which the average distance moved per hour formed the basis (Figure 2). Using the average distance moved per hour the skewness, RMSE and autocorrelation of (deviations in) activity were calculated. Furthermore, the entropy of activity was calculated using the raw RFID data. Each of these activity calculations is described in more detail below. Extreme outliers in the data were identified using a threshold of four times the standard deviation. Outliers were determined within rounds for the 5 activity descriptors. All outliers were identified using all animals in that round and any outliers observed were set to missing.

#### Mean Distance

The distance calculations were based on the registered antennas over time for each individual and day in the RFID log file. The center points of the antennas were used as an approximation of the location of birds within the antenna range, to calculate approximate distances moved. For a more elaborate description of how distances were calculated from the recorded antenna positions over time, see Van der Sluis et al. (2020). The total distance recorded was then divided by the recording duration between 07.00 and 23.00 for that specific day, to obtain a daily average distance moved per hour (**DADM**). This was done to allow for comparisons between days and rounds, even when data were missing for part of a day due to for example weighing of the birds. The mean distance moved (**MD**; presented in meters per hour) was calculated for each animal by taking the mean of the DADM in the first 2 wk after hatching, excluding days when data were missing for an individual.

#### Skewness

Skewness is a measure of the asymmetry of a distribution, with a positive skew meaning that the right tail of the distribution is longer than the left tail, and a negative skew meaning that the left tail is longer than the right tail (Legendre, 2012). For each individual, the skewness of the activity level, based on the DADM, was calculated using the e1071 package (Mayer et al., 2021) in R. Days when data were missing were excluded on an individual basis. For one bird, there were too few days available to calculate skewness and this bird was therefore excluded (Figure 1). The resulting output was a skewness value per individual (**Skew**).

#### Root Mean Square Error

The RMSE is a measure of the differences between model-predicted values and observed values. Given the expected decreasing trend in activity over time (e.g., van der Sluis et al. (2019)), linear regression models were used to obtain predicted activity levels over time for each individual's own linear pattern. To this end, linear models were fitted for each individual, with DADM modelled as a function of the day in the trial (i.e., age). Missing days of data were excluded on an individual basis. The differences between the predicted activity levels and the observed activity levels across the first 2 wk of life were used to obtain the RMSE of activity for each individual, with exclusion of missing values, that is, days on which no activity records were available. This was done using the following calculation:

$$RMSE_{j}=\sqrt{\frac{1}{n_{j}}\sum_{i=1}^{n_{j}}{\left(x_{p_{ij}}-x_{o_{ij}}\right)}^{2}}$$

where *RMSE_j_* represents the RMSE of activity for the *j^th^* individual, *n_j_* is the number of observations for the *j^th^* individual, $x_{p_{ij}}$ is the predicted observation *i* of the *j^th^* individual, and $x_{o_{ij}}$ is the observed observation *i* of the *j^th^* individual. The resulting output was an RMSE value per individual.

#### Autocorrelation

Using the residuals from the linear models described for the RMSE calculations, the lag-1 autocorrelation of deviations in activity was calculated for every individual. The lag-1 autocorrelation is the degree of correlation between the time series and the same time series set off by one time unit. Missing values for activity deviations were excluded, only complete cases per individual (that is, both the current deviation and the one-time-unit-offset deviation were available) were included in the calculation. The resulting output was an autocorrelation (**AC**) value per individual.

#### Entropy

Entropy is a measure of predictability and there are different statistical approaches to entropy. Here, sample entropy (**SampEn**) was used, which is a measure of the randomness or regularity of time series based on the existence of patterns (Delgado-Bonal and Marshak, 2019). Lower SampEn values indicate regularity and higher SampEn values indicate randomness. Here, entropy values were calculated for each individual and day, between 07.00 and 23.00. To this end, it was first determined for every minute whether an antenna switch was registered within this minute for this animal. Then, for each 15-min bin the number of minutes in which an antenna switch was registered was calculated. These values were then categorized into four classes based on the quantiles observed in the data (i.e., based on the range and distribution of active minutes), with 1 = very inactive (0–2 min), 2 = inactive (3–4 min), 3 = active (5–7 min), and 4 = very active (8–15 min). These classes for the 15-min bins across the day were then used as the underlying time series for the entropy calculation. The entropy values can therefore be interpreted as a measure of regularity in activity within days. However, entropy could only be accurately calculated for days without missing data. Therefore, entropy was only calculated on a subset of data of all birds in rounds 2, 3, 4 and 5. Round 1 (n = 64) was excluded, because of the earlier-mentioned missing data due to technical difficulties. Furthermore, days on which the birds were weighed or their leg bands were checked, were excluded. This resulted in 1,827 days of data available, from a total of 254 birds, for entropy calculation. The SampEn values were determined using the sample_entropy() function from the pracma package (Borchers, 2021) in R. The mean entropy per individual was then calculated for the 2-wk period (excluding missing values) and the resulting output was an entropy value per individual (**ENT**).

### Statistical Analyses

All analyses were performed using R version 4.0.2 (R Core Team, 2020). Descriptive statistics were used to examine the ADG and activity descriptor values for each of the tracking rounds. Moreover, the upper and lower quartiles of animals in terms of their ADG were determined, with an upper threshold of 72.27 g for the low ADG group (n = 80, with n = 14, 22, 10, 12 and 22 from rounds 1 to 5, respectively) and a lower threshold of 84.80 g for the high ADG group (n = 80, with n = 16, 8, 21, 16 and 19 from rounds 1 to 5, respectively), and descriptive statistics were also used to examine the activity descriptor values for these 2 groups. Wilcoxon rank sum tests were used to compare the activity descriptors between these 2 groups, as the descriptors were not normally distributed. Correlations between activity descriptors and ADG were determined using Kendall rank correlations, as ADG and several of the activity descriptors were not normally distributed and there were ties in the data, that is, multiple observations with the same value. For these correlations 95% CIs were determined using bootstrapping with the NSM3 package (Schneider et al., 2021). To take into account the confounding effects of round in which the birds were tracked and start body weight of the birds, a linear model with sum-to-zero contrasts was implemented. As no entropy data were available for round 1, the model only included rounds 2 to 5. To examine whether activity descriptors were correlated, pairwise Kendall's rank correlations were determined between descriptors (Supplementary data 1). From this, it became apparent that there was a strong correlation between MD and RMSE (tau = 0.32 [95% CI 0.26–0.38], *P* < 0.001). As RMSE was already observed to be correlated with ADG (see Results section), RMSE was included in the model for estimating ADG, and MD was not. Round, start body weight and the activity descriptors apart from MD were included as fixed effects. To test the fixed effects, a backward stepwise approach without interactions was used that included all these effects. The resulting terms that were left were all included in two-way interactions. Backward selection was then again performed. The resulting final model was

$$Y_{ijk}=\mu +Round_{i}+\beta {\left(SW\right)}_{j}+\beta {\left(RMSE\right)}_{k}+e_{ijk}$$

where $Y_{ijk}$ is the average daily gain, μ is the overall mean, $Round_{i}$ is the round of tracking (i= 2-5), $\beta {\left(SW\right)}_{j}$ is the start weight, $\beta {\left(RMSE\right)}_{k}$ is the RMSE and $e_{ijk}$ is the residual term. No obvious deviations from normality or homoscedasticity were observed upon visual inspection of the residuals of the model. The ggplot2 (Wickham, 2016) package was used to make the visualizations. The level of statistical significance was set at 0.05 and in the text reported results are rounded to 2 decimals.

## RESULTS

### Average Daily Gain

The mean ADG across all rounds was 77.46 (SD 10.48) grams. The ADG was very similar for the different rounds, with 78.04 (SD 7.80) grams for round 1, 74.81 (SD 11.13) grams for round 2, 79.93 (SD 10.89) grams for round 3, 78.86 (SD 8.24) grams for round 4, and 75.91 (SD 13.08) grams for round 5.

### Activity Descriptors

**Figure 3:**
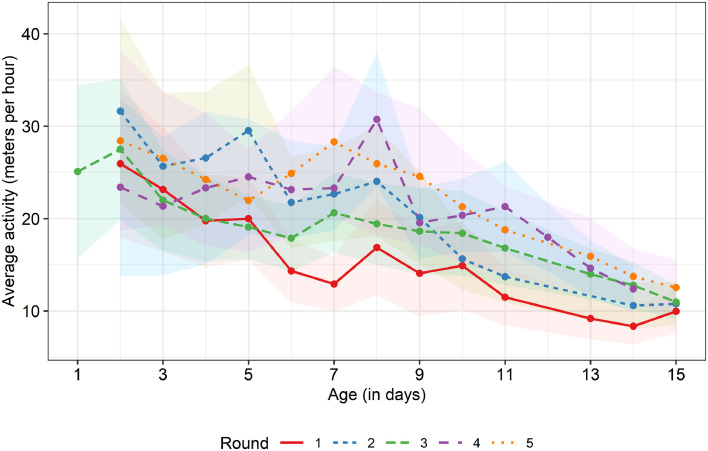
Average activity level over time for the different rounds. Shaded areas indicate SD ranges.

**Figure 4:**
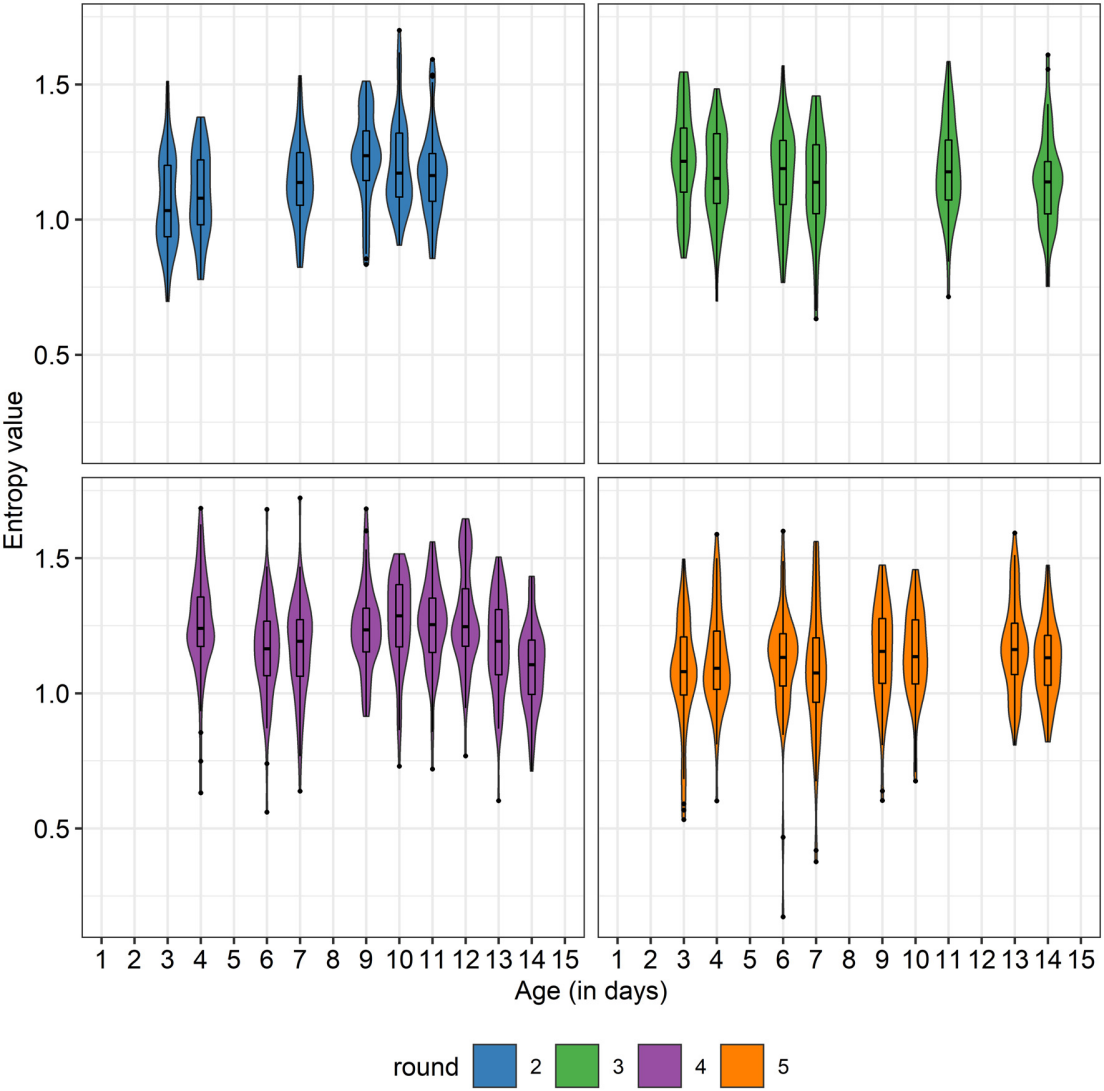
Entropy of daily activity over time for each of the four included rounds. Violin plots and boxplots represent the distribution of the entropy values within a day. Different colors represent different rounds.

The daily activity levels across the first 2 wk after hatching are shown in Figure 3. Even though the rounds differed in their average activity level and exact pattern, a decrease in activity over time was observed in all rounds. The mean recorded distance was relatively low in round 1, likely due to the earlier-mentioned wet bedding. The entropy of daily activity over time for each of the 4 included rounds is shown in Figure 4. The average entropy remained relatively stable over time and the different rounds did not show large differences. However, there was quite some variation in individual entropy values within days and rounds (Figure 4). The mean values for all activity descriptors are shown in Table 2 (see Supplementary data 2 and 3 for the distributions of the activity descriptors and a Principal Component Analysis of the activity descriptors, respectively. The PCA suggested that the first dimension is linked to the mean activity and occurrence of deviations in activity and that the second dimension is linked more to the duration and direction of the deviations). The Skew, RMSE, AC, and ENT values did not show large differences between rounds. There was variation in MD values between rounds, as was already shown in Figure 3. The average MD ranged between 15.21 (round 1) and 22.11 (round 5) meters per hour. There were no statistically significant differences in mean values for most of the activity descriptors for the upper and lower quartiles of animals in terms of ADG, except for the average MD and RMSE, which were both higher for the low ADG group (W = 2501, *P* = 0.017 and W = 2526, *P* = 0.022, respectively).

**Table 2:** Mean values for the activity descriptors across all rounds, per round and for the lower and upper quartile of animals based on average daily gain.

Activity descriptor	Overall	Round 1	Round 2	Round 3	Round 4	Round 5	Lower quartile ADG	Upper quartile ADG
Mean distance (in meters/hour/day)	19.74 (4.63)	15.21 (3.21)	21.00 (3.81)	18.78 (2.98)	21.24 (3.91)	22.11 (5.11)	20.88 (4.95)	19.09 (4.03)
Skewness	0.37 (0.66)	0.75 (0.58)	0.17 (0.63)	0.52 (0.65)	0.35 (0.65)	0.08 (0.58)	0.25 (0.56)	0.41 (0.73)
Root mean square error	3.65 (1.62)	3.00 (1.21)	3.74 (1.42)	3.16 (1.66)	4.48 (1.58)	3.71 (1.76)	3.94 (1.83)	3.21 (1.17)
Autocorrelation	0.18 (0.31)	0.16 (0.32)	0.10 (0.26)	0.25 (0.32)	0.14 (0.24)	0.26 (0.35)	0.21 (0.30)	0.17 (0.29)
Entropy	1.16 (0.09)	NA^1^	1.15 (0.08)	1.17 (0.07)	1.21 (0.07)	1.12 (0.09)	1.15 (0.09)	1.16 (0.08)

Standard deviations are indicated between parentheses.

1 Entropy was not calculated for round 1 due to too many missing data.

### Correlations Between Activity Descriptors and ADG

Table 3 shows the rank correlations between the activity descriptors and ADG, when combining the data from all 5 rounds, with exception of entropy where round 1 was excluded. A negative correlation between the root mean square error and ADG was observed, indicating that broilers with a higher root mean square error have a lower ADG. A trend for a negative correlation between the mean distance and ADG was also observed, suggesting that birds that walk longer distances have a lower ADG.

**Table 3:** Rank correlations between the activity descriptors and average daily gain.

Activity descriptor	tau	95% CI	z value	*P* value
Mean distance	−0.065	−0.131 to 0.004	−1.724	0.085
Skewness	0.016	−0.069 to 0.097	0.426	0.670
Root mean square error	−0.105	−0.176 to −0.034	−2.787	0.005
Autocorrelation	−0.018	−0.092 to 0.055	−0.486	0.627
Entropy^1^	0.024	−0.064 to 0.117	0.564	0.573

1 Entropy was not calculated for round 1 due to too many missing data and was therefore excluded here.

### Estimating ADG Using Activity Descriptors

To take into account the possible confounding effects of round and start body weight, a linear model was implemented. The results from this model are presented in Table 4. In this table, the sum-to-zero contrasts are presented, meaning that, for example, round 2 had an estimated 2.59 g/day lower ADG than the overall average ADG across all rounds. The model had an adjusted R^2^ of 0.08. Overall, there were differences between rounds in ADG and birds with higher start weights showed a higher ADG (estimate = 0.41, *P* = 0.005). The ADG decreased when the RMSE increased (estimate = −1.08, *P* = 0.011). An increase in RMSE means that the activity of an individual deviates more, in both directions (higher and lower), from a linear pattern over time.

**Table 4:** Results of the linear model for the predicted average daily gain (grams per day).

Factor	df	F-value	Pr (>F)	Estimate	SE	t-value	Pr (>|t|)
Intercept	1	51.480	<0.001	59.481	8.290	7.175	<0.001
Round	3	3.874	0.010				
Round 2				−2.594	1.165	−2.228	0.027
Round 3				3.024	1.325	2.282	0.023
Round 4				1.532	1.192	1.285	0.200
Bodyweight start	1	7.876	0.005	0.414	0.147	2.806	0.005
Root mean square error	1	6.493	0.011	−1.078	0.423	−2.548	0.011

## DISCUSSION

In this study, the relationship between early activity and body weight gain was investigated. Activity records, collected using an RFID system and converted to several dynamic descriptors of activity, and body weight records were combined. The expectations were that a lower ADG would be linked to: 1) a lower mean activity; 2) a reduced (due to consistently low activity) or increased (due to some uncharacteristically inactive days) skewness of activity; 3) an increased RMSE due to more deviations in activity levels; 4) an increased autocorrelation of activity deviations due to activity deviations on subsequent days becoming more related; and 5) an increased entropy of activity due to a less regular daily activity pattern. This was based on the assumption that a lower ADG is indicative of reduced welfare. It must be noted, however, that this may have been an oversimplification and that the relationship between ADG, activity and welfare is unclear and may have effects in several directions. In terms of the effect of activity on ADG and welfare, it could be broilers that are more active might have improved welfare, or at least seem to not be limited in their activity by leg health problems for example, and possibly a lower ADG due to the higher energy expenditure. On the other hand one might expect that birds that are not doing well show a reduced growth, for example as a consequence of chronic stress (shown using corticosterone treatment; Wang et al., 2013), and that therefore a lower ADG may be indicative of reduced welfare. These different and possibly simultaneously acting effects are difficult to disentangle based on the current data, and require more research. Overall, only a few of the descriptors of activity were observed to be linked to ADG and these did not explain a large amount of the variance in ADG. The main findings will be elaborated on in the remainder of this discussion.

### Average Daily Gain

The ADG was similar in all 5 production rounds. The overall mean ADG observed in this study was 77.46 (SD 10.48) g, which is higher than the growth reported for conventional broiler production in the Netherlands (i.e., a final body weight of 2,542 g on d 41; van Horne, 2020). This difference is likely caused by the fact that pure lines and only males were studied here and that the housing density in this study was relatively low compared to commercial practice. However, our mean ADG does not differ substantially from other reports in literature, as for example Meyer et al. (2019) reported ADGs of 66.6 and 72.3 g for broilers in a non-enriched or laser-enriched environment.

### Activity Descriptors

Only RMSE showed a statistically significant correlation with ADG, all other activity descriptors did not. The correlation between RMSE and ADG was not strong, but RMSE was still shown to be linked to ADG even when taking production round and start weight into account. Each of the activity descriptors is discussed in more detail here.

#### Mean Distance

Overall, a decrease in mean distance moved over time was observed in this study. This decrease in activity over time matches with earlier observations and reports in literature (Weeks et al., 2000; van der Sluis et al., 2019), and is likely linked to the birds’ increasing body weights (Tickle et al., 2018). At the same time, there appeared to be more variation in activity in the first days compared to later in the 2-wk period under study (Figure 3). This highlights the importance of activity records early in life, as later in life there might be too little variation in activity to really be able to distinguish individuals (see also Ellen et al. (*unpublished data*), who used a dataset partly overlapping with the current study). No statistically significant correlation between mean distance and ADG was observed, but there was a trend suggesting that the more active birds had a lower ADG. Moreover, when comparing the low and high ADG quartile groups, the average MD was higher for the low ADG group. This indication fits with our earlier observations of lower activity levels for heavier birds (van der Sluis et al., 2019), as we assumed that birds with higher body weights later in life would have a higher ADG than lighter birds. This is supported by the observation of a strong positive correlation between ADG and final body weight in this study (tau = 0.80 [95% CI 0.78–0.83]). However, other studies have observed that differences in body weight are more pronounced later in life. For example, Dixon (2020) observed that broilers with different growth rates showed no difference in proportion of observations in which locomotion was observed when the birds were young and did not differ significantly in their body weight at 2-wk-old, but a difference in locomotory activity was observed from 30 days old onward and in body weight from 4-wk-old onwards. One potential hypothesis for why we see this relationship between ADG and activity already early in life is that the overall ADG is strongly affected by the weight gain in the first two weeks. This weight gain might be related to early activity, and this could for example be the case when the ADG later in life is very similar for all birds and differences in ADG occur mainly early in the growing period. Alternatively, ADG and activity may not be directly linked, but may both be outcomes of birds’ behavioral characteristics, and the interindividual variation therein, in a broader sense. It has been shown that broilers may differ in their responses in a T-maze (Jones et al., 1999; Marin et al., 2003), where they can reinstate visual contact with conspecifics, and that birds that have short latencies to exit the start box or reach the mirror fast, show higher body weights later in life (at 42 and 56 days old) than birds that are slower (Marin et al., 2003). It is thought that individual variation in sociality plays an important role in the observed T-maze response (Jones et al., 1999) and Marin et al. (2003) hypothesize that this variation in sociality may affect the welfare and productivity of the birds. As Marin et al. (2003) suggest, the birds that are fast in the T-maze might be more sociable and may therefore be better able to cope with the housing in large groups that is common in practice. This might (partially) underpin the higher body weight of these birds compared to birds that are slower in the T-maze (Marin et al., 2003), as chronic stress in broilers may result in reduced body weights (as shown using corticosterone treatment; Wang et al., 2013). It has furthermore been observed that chronic stress in broilers, simulated through corticosterone administration, increases the percentage of time that they walk (Wang et al., 2013). This could also explain the observation of birds with a lower ADG walking longer distances in the current study. A limitation of this study is that only ADG across the full growing period was examined, as a starting point for examining the relationship between early activity and body weight gain, and thus approaching the growth of birds as linear over time. In reality, ADG is not constant across the growing period (e.g., Zuidhof et al., (2014)) and there might be nuances to this growth pattern that other curves describe better (Topal and Bolukbasi, 2008). Individual growth curves would therefore be interesting to consider in future research, to examine which parts of the growth curve are related to different activity descriptors.

#### Skewness

For all rounds the average skewness of activity was close to zero, indicating fairly symmetrical distributions of daily activity levels during the 2-wk period under study. No correlations between skewness of activity and ADG were observed. Other studies have examined skewness in relation to resilience (e.g., Berghof et al., 2019a; van der Zande et al., 2020). Disturbances may result in stress and, given that broilers experiencing chronic stress may have reduced body weights (Wang et al., 2013), this may be reflected in the ADG. Therefore, even though no additional challenges were implemented in the current study, the concept of resilience may provide interesting insights. For animals that are resilient, skewness around zero would be expected and indeed for pigs it was observed that a decrease in skewness, towards zero, after a health challenge (that is, an infection with the Porcine Reproductive and Respiratory Syndrome Virus; **PRRSV**) lowered the risk of mortality (van der Zande et al., 2020). It must however be noted that skewness was not found to be related to morbidity. In accordance, other studies have concluded that skewness is not the most promising dynamic descriptor, for example, for body weight deviations of layer chickens in relation to resilience (Berghof et al., 2019a). Here it appears that also for ADG in broilers the skewness, in this case of the activity level, is not informative. However, given that no additional challenges were implemented in our study, it could be that any perceived challenges were too moderate to result in large differences in skewness of activity. When more challenging conditions are presented, potentially the skewness of activity may be informative for ADG in broilers, but this remains to be investigated.

#### Root Mean Square Error

Across the different rounds, the average RMSE of activity was quite similar. A negative relationship with ADG was observed, also when taking production round and start weight into account. This relationship indicated that broilers with a higher RMSE, that is, more deviations from the expected linear trend in activity, had a lower ADG. In other words, it appears that when broilers strongly fluctuate in their activity level, instead of showing a steady, and generally declining, activity level over time, their growth is reduced. Other studies examining the RMSE of activity are limited, and mainly performed under health challenging conditions, but Van der Zande et al. (2020) observed that a higher RMSE after a PRRSV challenge tended to increase the risk of morbidity in pigs. In a study examining the RMSE of feed intake or feeding duration in pigs under a natural disease challenge, Putz et al. (2019) observed positive genetic correlations between both RMSEs and mortality, and a negative genetic correlation between the RMSE of feed intake and finishing ADG, which is in line with our observation of an increased RMSE (albeit of activity) being linked to a reduced performance. Although RMSE was observed to positively correlate with MD (Supplementary data 1), RMSE might be a more sensitive indicator of differences in activity patterns than mean activity levels, as mean activity levels alone cannot distinguish between a generally very active individual with several short, major activity decreases and a consistently moderately active individual. However, a difficulty with RMSE values is that they do not distinguish between different directions of deviations: high RMSE values could indicate 1) some days with higher activity levels than expected or predicted, 2) some days with lower activity levels than expected or predicted, or 3) a combination of both. Consequently, it is difficult to interpret the behavior underlying the association between RMSE and reduced ADG. We hypothesize that one possibility could be that activity levels are linked to the number of feeder visits in broilers, and hereby potentially to feed intake. If there are fluctuations in activity, this may be indicative of fluctuations in feeding motivation (or feeding) and this may negatively affect the ADG, in line with the earlier-mentioned observation of a negative genetic correlation between the RMSE of feed intake and finishing ADG in pigs by Putz et al. (2019). However, more research is required to test this hypothesis through, for example, provisioning of fluctuating amounts of feed to broilers, or to examine whether other factors are at play that affect both ADG and activity.

#### Autocorrelation

The mean observed (lag-1 day) autocorrelations of deviations in activity for the different rounds were all close to zero. This suggests that deviations in activity on subsequent days are unrelated (Berghof et al., 2019b). Although discussed for other traits than locomotor activity, Berghof et al. (2019b) note that a lag-1 autocorrelation of deviations around zero is expected for individuals that show no disturbances or that recover fast from disturbances. It appears that, on average, the animals in our study showed few deviations in their daily activity level or, if they did, those changes did not last for prolonged periods of time. This is not surprising, as no additional challenges were implemented in our study. We observed no correlation with ADG. Generally, it is expected that less resilient animals show a positive autocorrelation of deviations (Berghof et al., 2019b). However, studies have observed that autocorrelation was not informative, for example for morbidity or mortality in pigs, where autocorrelation in activity was calculated (van der Zande et al., 2020). It appears that also for ADG in broilers the lag-1 autocorrelation of deviations in activity is not informative.

#### Entropy

Studies have indicated that entropy can be informative, or even predictive, of human behavior, health, and well-being (Glenn et al., 2006; Montirosso et al., 2010; Okamoto et al., 2022). Entropy in non-human animal behavior has not been extensively studied, but there are indications that entropy can be an informative measure of non-human animal behavior as well (e.g., Stamps et al., 2013; Guerrero-Bosagna et al., 2020). For example, McVey et al. (2020) studied milking order in dairy cattle using entropy, and observed that cows at the front and rear of the queue were more consistent in their entry position than individuals in the middle of the queue. Eguiraun et al. (2014) studied the collective response in groups of fish to a stochastic event (sudden hit in the tank) through entropy and observed that a group of fish exposed to a contaminant (methylmercury) showed lower entropy compared to control groups. In the current study, the different production rounds were observed to show similar entropy means. No correlation between entropy and ADG was observed, indicating that more or less regular patterns in daily activity early in life are not associated with differences in ADG. However, it would be worth exploring different ways of applying entropy in the future to animal behavior data to understand how to best capture the behavior feature of interest.

### Predicting ADG Using Activity Descriptors and Future Directions

This study provides indications that deviations in early activity, represented in the RMSE of activity, are linked to a decreased ADG. However, the relationships were not strong and the implemented model explained little of the total variation in ADG in broilers (8%). This was expected, as ADG in broilers is known to be affected by many factors that likely play a larger role in the observed ADG than activity does. For example, body weight gain can be affected by the type of feed that is provided, the amount of feed consumed and the feed conversion ratio (Havenstein et al., 2003; Marchesi et al., 2021), as well as management factors such as litter treatment (de Toledo et al., 2020). For practice this means that the RMSE of activity is insufficient to fully distinguish between birds with high or low ADG, as these other factors also play (likely even larger) roles in the observed ADG. For future research, it is recommended to also record these factors at the individual level, and to model growth curves instead of overall ADG to obtain a more complete picture of broiler growth and the factors that play a (predictive) role. In this way, more subtle patterns in growth can perhaps be detected and linked to activity in broilers. Besides the observations of RMSE and production round being linked to ADG, start weight was also observed to affect ADG. Broilers with a higher start weight showed a higher ADG, and the ADG was strongly correlated with the end weight in our study. Other studies have also observed a correlation between start weight and final body weight. For example, Willemsen et al. (2008) examined the predictive value of several chick quality measurements for slaughter-age body weight in different breeder lines and observed positive correlations between the body weight at 1-day-old and the body weight on d 42. It must be noted, however, that several other studies observed no correlation between start weight and body weight later in life (e.g., Pinchasov, (1991)).

There was no strong and significant correlation between the distance moved early in life and ADG. Similar results have been reported by Ruiz-Feria et al. (2014), who observed that an increased distance between water and feed did not reduce the body weight of broilers, and similar observations have been reported by Reiter and Bessei (2009). However, addition of a ramp between water and feed did reduce the body weight of broilers, and this was suggested to be due to avoidance of the ramp as the broilers grew heavier, as the birds with a ramp ate less (Ruiz-Feria et al., 2014). This highlights the importance of examining the method for increasing activity closely, to assess whether there are no unintentional side effects of increasing the activity level of broilers. Overall, the current study suggests that it is possible to increase early life activity without necessarily negatively affecting broiler growth. Increased activity can positively affect leg health in broilers (Reiter and Bessei, 2009; Kaukonen et al., 2017), and hereby contribute to improved broiler welfare and broiler production economics.

Not all activity descriptors examined in this study were linked to ADG in broilers. However, the continuous data on activity and subsequently calculated activity descriptors can provide us with more insight into the activity patterns of individual broilers over time. Possibly, this type of information can be informative or predictive for other traits in broilers, such as leg health. Given the individual variation that was observed in for example entropy of activity, there appears to be potential for further examination of whether and how such differences relate to different traits in broilers, to in the future be better able to monitor or even predict broiler health and welfare. Especially when the conditions in which the broilers are kept in future studies are more challenging than they were here, differences in activity patterns may be more pronounced.

## CONCLUSIONS

This study examined the relationship between RFID-recorded early life activity patterns and body weight gain in broilers. The RMSE of activity was correlated with ADG, and suggested that broilers with a higher RMSE had a lower ADG, but currently explained only a small part of the variation in ADG. Overall, this study suggests that increasing early life activity without negatively affecting body weight gain in broilers is feasible, as there were no strong and statistically significant correlations between ADG and distances moved early in life. Through the expected positive effects of increased activity on leg health, this may in the future contribute to improved broiler health and welfare. Moreover, the activity descriptors studied here can provide more insight into the activity patterns of individual broilers over time, and allow for further examination of whether and how such patterns relate to different traits in broilers, to in the future be better able to monitor or even predict broiler health and welfare.

## Appendix Supplementary materials

Image, application 1

Image, application 2

Image, application 3

## Footnotes

Supplementary material associated with this article can be found in the online version at doi:10.1016/j.psj.2022.102086.

## References

[bib0001] Asher L., Collins L.M., Ortiz-Pelaez A., Drewe J.A., Nicol C.J., Pfeiffer D.U. (2009). Recent advances in the analysis of behavioural organization and interpretation as indicators of animal welfare. J. R. Soc. Interface.

[bib0002] Berghof T.V.L., Bovenhuis H., Mulder H.A. (2019). Body weight deviations as indicator for resilience in layer chickens. Front. Genet..

[bib0003] Berghof T.V.L., Poppe M., Mulder H.A. (2019). Opportunities to improve resilience in animal breeding programs. Front. Genet..

[bib0004] Bessei W. (2006). Welfare of broilers: a review. Worlds Poult. Sci. J..

[bib0005] Bizeray D., Leterrier C., Constantin P., Picard M., Faure J.M. (2000). Early locomotor behaviour in genetic stocks of chickens with different growth rates. Appl. Anim. Behav. Sci..

[bib0006] Bizeray D., Leterrier C., Constantin P., Picard M., Faure J.M. (2002). Sequential feeding can increase activity and improve gait score in meat-type chickens. Poult. Sci..

[bib0007] Bokkers E.A.M., Koene P. (2003). Behaviour of fast- and slow growing broilers to 12 weeks of age and the physical consequences. Appl. Anim. Behav. Sci..

[bib0008] Bokkers E.A.M., Zimmerman P.H., Rodenburg T.B., Koene P. (2007). Walking behaviour of heavy and light broilers in an operant runway test with varying durations of feed deprivation and feed access. Appl. Anim. Behav. Sci..

[bib0009] Borchers, H. W. 2021. Pracma: practical numerical math functions. R package version 2.3.3. Accessed Nov, 2021. https://CRAN.R-project.org/package=pracma

[bib0010] Butterworth A. (1999). Infectious components of broiler lameness: a review. Worlds Poult. Sci. J..

[bib0011] Cobb. 2018. Broiler management guide. Accessed Aug, 2019.https://cobbstorage.blob.core.windows.net/guides/5fc96620-0aba-11e9-9c88-c51e407c53ab.

[bib0012] Danbury T.C., Weeks C.A., Chambers J.P., Waterman-Pearson A.E., Kestin S.C. (2000). Self-selection of the analgesic drug carprofen by lame broiler chickens. Vet. Rec..

[bib0013] Dawkins M.S., Cain R., Roberts S.J. (2012). Optical flow, flock behaviour and chicken welfare. Anim. Behav..

[bib0014] de Toledo T.D.S., Roll A.A.P., Rutz F., Dallmann H.M., Dai Pra M.A., Leite F.P.L., Roll V.F.B. (2020). An assessment of the impacts of litter treatments on the litter quality and broiler performance: a systematic review and meta-analysis. PLoS One.

[bib0015] Delgado-Bonal A., Marshak A. (2019). Approximate entropy and sample entropy: a comprehensive tutorial. Entropy..

[bib0016] Dixon L.M. (2020). Slow and steady wins the race: the behaviour and welfare of commercial faster growing broiler breeds compared to a commercial slower growing breed. PloS One.

[bib0017] Eguiraun H., López-de-Ipiña K., Martinez I. (2014). Application of entropy and fractal dimension analyses to the pattern recognition of contaminated fish responses in aquaculture. Entropy..

[bib0018] Fasmer O.B., Johansen E.B. (2016). Patterns of motor activity in spontaneously hypertensive rats compared to Wistar Kyoto rats. Behav. Brain Funct..

[bib0019] Glenn T., Whybrow P.C., Rasgon N., Grof P., Alda M., Baethge C., Bauer M. (2006). Approximate entropy of self-reported mood prior to eposides in bipolar disorder. Bipolar Disord..

[bib0020] Guerrero-Bosagna C., Pértille F., Gomez Y., Rezaei S., Gebhardt-Henrich S.G., Vögeli S., Stratmann A., Voelkl B., Toscano M.J. (2020). DNA methylation variation in the brain of laying hens in relation to differential behavioral patterns. Comp. Biochem. Physiol. - D: Genom. Proteom..

[bib0021] Havenstein G.B., Ferket P.R., Qureshi M.A. (2003). Growth, livability, and feed conversion of 1957 versus 2001 broilers when fed representative 1957 and 2001 broiler diets. Poult. Sci..

[bib0022] Jones R.B., Marin R.H., Garcia D.A., Arce A. (1999). T-maze behaviour in domestic chicks: a search for underlying variables. Anim. Behav..

[bib0023] Kaukonen E., Norring M., Valros A. (2017). Perches and elevated platforms in commercial broiler farms: use and effect on walking ability, incidence of tibial dyschondroplasia and bone mineral content. Animal.

[bib0024] Kestin S.C., Knowles T.G., Tinch A.E., Gregory N.G. (1992). Prevalence of leg weakness in broiler chickens and its relationship with genotype. Vet. Rec..

[bib0025] Legendre P., Legendre P., Legendre L. (2012). Numerical Ecology.

[bib0026] Marchesi J.A.P., Ono R.K., Cantão M.E., Ibelli A.M.G., Peixoto J.de O., Moreira G.C.M., Godoy T.F., Coutinho L.L., Munari D.P., Ledur M.C. (2021). Exploring the genetic architecture of feed efficiency traits in chickens. Sci. Rep..

[bib0027] Marin R.H., Satterlee D.G., Castille S.A., Jones R.B. (2003). Early T-maze behavior and broiler growth. Poult. Sci..

[bib0028] McVey C., Hsieh F., Manriquez D., Pinedo P., Horback K. (2020). Mind the queue: a case study in visualizing heterogeneous behavioral patterns in livestock sensor data using unsupervised machine learning techniques. Front. Vet. Sci..

[bib0029] Meyer D., Dimitriadou E., Hornik K., Weingessel A. (2021). e1071: misc functions of the department of statistics, probability theory group (formerly: E1071), TU Wien. R package version 1.7-7. Accessed May, 2021. https://CRAN.R-project.org/package=e1071.

[bib0030] Meyer M.M., Johnson A.K., Bobeck E.A. (2019). A novel environmental enrichment device improved broiler performance without sacrificing bird physiological or environmental quality measures. Poult. Sci..

[bib0031] Montirosso R., Riccardi B., Molteni E., Borgatti R., Reni G. (2010). Infant's emotional variability associated to interactive stressful situation: a novel analysis approach with sample entropy and Lempel-Ziv complexity. Infant. Behav. Dev..

[bib0032] Nielsen B.L., Kjaer J.B., Friggens N.C. (2004). Temporal changes in activity measured by passive infrared detection (PID) of broiler strains growing at different rates. Arch. Geflügelk..

[bib0033] Okamoto S., Ishii M., Hibi S., Akishita M., Yamaguchi Y. (2022). Breathing irregularities before sleep onset on polysomnography in patients with heart diseases. Sleep Breath.

[bib0034] Pinchasov Y. (1991). Relationship between the weight of hatching eggs and subsequent early performance of broiler chicks. Br. Poult. Sci..

[bib0035] Putz A.M., Harding J.C.S., Dyck M.K., Fortin F., Plastow G.S., Dekkers J.C.M., PigGen Canada (2019). Novel resilience phenotypes using feed intake data from a natural disease challenge model in wean-to-finish pigs. Front. Genet..

[bib0036] R Core Team (2020).

[bib0037] Reiter K., Bessei W. (2009). Effect of locomotor activity on leg disorder in fattening chicken. Berl. Munch. Tierarztl. Wochenschr..

[bib0038] Ruiz-Feria C.A., Arroyo-Villegas J.J., Pro-Martinez A., Bautista-Ortega J., Cortes-Cuevas A., Narciso-Gaytan C., Hernandez-Cazares A., Gallegos-Sanchez J. (2014). Effects of distance and barriers between resources on bone and tendon strength and productive performance of broiler chickens. Poult. Sci..

[bib0039] Rutherford K.M.D., Haskell M.J., Glasbey C., Jones R.B., Lawrence A.B. (2003). Detrended fluctuation analysis of behavioural responses to mild acute stressors in domestic hens. Appl. Anim. Behav. Sci..

[bib0040] Sanchez-Rodriguez E., Benavides-Reyes C., Torres C., Dominguez-Gasca N., Garcia-Ruiz A.I., Gonzalez-Lopez S., Rodriguez-Navarro A.B. (2019). Changes with age (from 0 to 37 D) in tibiae bone mineralization, chemical composition and structural organization in broiler chickens. Poult. Sci..

[bib0041] Schneider, G., E. Chicken, and R. Becvarik. 2021. NSM3: Functions and Datasets to Accompany Hollander, Wolfe, and Chicken - Nonparametric Statistical Methods, 3rd edition. R package version 1.16. Accessed Apr, 2021. https://CRAN.R-project.org/package=NSM3

[bib0042] Stamps J.A., Saltz J.B., Krishnan V.V. (2013). Genotypic differences in behavioural entropy: unpredictable genotypes are composed of unpredictable individuals. Anim. Behav..

[bib0043] Tickle P.G., Hutchinson J.R., Codd J.R. (2018). Energy allocation and behaviour in the growing broiler chicken. Sci. Rep..

[bib0044] Topal M., Bolukbasi Ş.C. (2008). Comparison of nonlinear growth curve models in broiler chickens. J. Appl. Anim. Res..

[bib0045] van der Sluis M., de Haas Y., de Klerk B., Rodenburg T.B., Ellen E.D. (2020). Assessing the activity of individual group-housed broilers throughout life using a passive radio frequency identification system - a validation study. Sensors.

[bib0046] van der Sluis M., de Klerk B., Ellen E.D., de Haas Y., Hijink T., Rodenburg T.B. (2019). Validation of an ultra-wideband tracking system for recording individual levels of activity in broilers. Animals.

[bib0047] van der Zande L.E., Dunkelberger J.R., Rodenburg T.B., Bolhuis J.E., Mathur P.K., Cairns W.J., Keyes M.C., Eggert J.M., Little E.A., Dee S.A., Knol E.F. (2020). Quantifying individual response to PRRSV using dynamic indicators of resilience based on activity. Front. Vet. Sci..

[bib0048] van Horne, P. L. M. 2020. Economics of broiler production systems in the Netherlands; economic aspects within the Greenwell sustainability assessment model. Wageningen, Wageningen Economic Research, Report 2020-027.

[bib0049] Vasdal G., Vas J., Newberry R.C., Moe R.O. (2019). Effects of environmental enrichment on activity and lameness in commercial broiler production. J. Appl. Anim. Welf. Sci..

[bib0050] Vestergaard K.S., Sanotra G.S. (1999). Relationships between leg disorders and changes in the behaviour of broiler chickens. Vet. Rec..

[bib0051] Wang S., Ni Y., Guo F., Fu W., Grossmann R., Zhao R. (2013). Effect of corticosterone on growth and welfare of broiler chickens showing long or short tonic immobility. Comp. Biochem. Physiol. Part A Mol. Integr. Physiol..

[bib0052] Weeks C.A., Danbury T.D., Davies H.C., Hunt P., Kestin S.C. (2000). The behaviour of broiler chickens and its modification by lameness. Appl. Anim. Behav. Sci..

[bib0053] Wickham H. (2016).

[bib0054] Willemsen H., Everaert N., Witters A., De Smit L., Debonne M., Verschuere F., Garain P., Berckmans D., Decuypere E., Bruggeman V. (2008). Critical assessment of chick quality measurements as an indicator of posthatch performance. Poult. Sci..

[bib0055] Zuidhof M.J., Schneider B.L., Carney V.L., Korver D.R., Robinson F.E. (2014). Growth, efficiency, and yield of commercial broilers from 1957, 1978, and 2005. Poult. Sci..

