# Supplementary material for: Early locomotor activity in broilers and the relationship with body weight gain
Source: Poult Sci. 2022 Jul 30;101(10):102086. doi: 10.1016/j.psj.2022.102086 (PMC9445389; doi:10.1016/j.psj.2022.102086)
Supplement: Supplementary file 1 [file mmc1.docx]

**Supplementary data 1**

Table S1.1: Kendall rank correlations between activity descriptors^1^. The following abbreviations are used: MD = mean distance, Skew = skewness, RMSE = root mean square error, AC = autocorrelation, and ENT = entropy.

|  | MD | Skew | RMSE | AC | ENT |
| --- | --- | --- | --- | --- | --- |
| MD |  | -0.17 (-0.24 – -0.10) | 0.32 (0.26 – 0.38) | 0.02 (-0.05 – 0.10) | -0.09 (-0.19 – -0.00) |
| Skew |  |  | 0.11 (0.04 – 0.19) | -0.05 (-0.13 – 0.02) | 0.03 (-0.07 – 0.11) |
| RMSE |  | Legend   \| ns \| p < 0.05 \| p < 0.01 \| p < 0.001 \| \| --- \| --- \| --- \| --- \| |  | 0.20 (0.12 – 0.28) | -0.04 (-0.13 – 0.05) |
| AC |  |  |  |  | -0.04 (-0.13 – 0.05) |
| ENT |  |  |  |  |  |

^1^ 95%-CIs are indicated between brackets.
