# Supplementary figures and images for: Early locomotor activity in broilers and the relationship with body weight gain
Source: Poult Sci. 2022 Jul 30;101(10):102086. doi: 10.1016/j.psj.2022.102086 (PMC9445389; doi:10.1016/j.psj.2022.102086)

**Supplementary data 2**

**
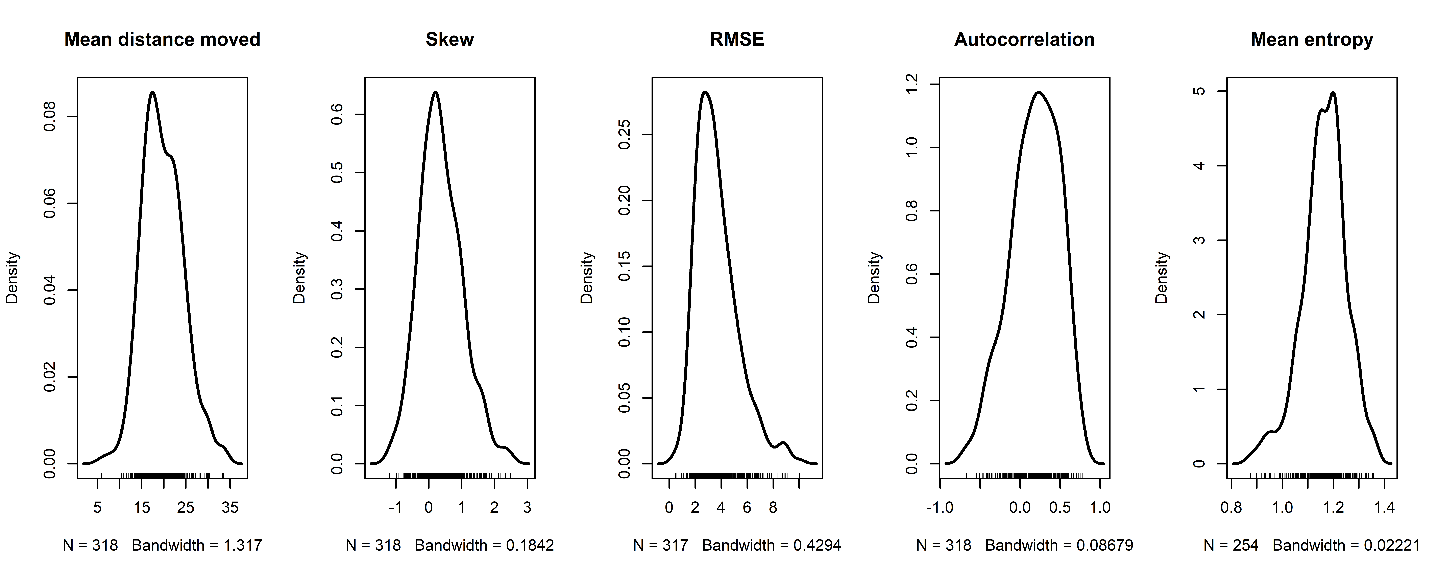
**

Figure S2.1: Density distributions of the activity descriptors.

Supplement: Supplementary file 2 [file mmc2.docx]
