# Supplementary material for: Early locomotor activity in broilers and the relationship with body weight gain
Source: Poult Sci. 2022 Jul 30;101(10):102086. doi: 10.1016/j.psj.2022.102086 (PMC9445389; doi:10.1016/j.psj.2022.102086)
Supplement: Supplementary file 3 [file mmc3.docx]

**Supplementary data 3**

A Principal Component Analysis (PCA) was performed on the activity descriptors, including only those birds that had all data available (n = 254), using the prcomp() function in R. Figure S3.1 shows a visualization of the PCA results, made using the factoextra package (Kassambara and Mundt, 2020) in R. The RMSE of activity had the largest contribution to the first dimension, followed by the mean distance moved. Moreover, regarding this first dimension, the PCA suggests a correlation between RMSE and mean distance, which is supported by the observed positive Kendall rank correlation between these two variables of 0.32 (95%-CI 0.26 – 0.38; see Supplementary data 1), and between mean distance and mean entropy, which is supported by the observed negative Kendall rank correlation of -0.09 (95%-CI -0.19 – -0.00; see Supplementary data 1). The skewness of activity had the largest contribution to the second dimension of the PCA. The first dimension of the PCA appears to be linked to the average activity of birds and the occurrence of deviations therein. The mean distance informs us about the average activity of the birds, the RMSE informs us about the overall degree of deviations in activity (but not the direction therein) and the mean entropy tells us how regular the activity pattern is within days. The second dimension appears to be linked more to the duration and direction of the deviations, with autocorrelation telling us whether the deviations last long and whether deviations are overcompensated (e.g., after a day of lower-than-average activity a day of higher-than-average activity), and the skewness telling us whether the majority of daily activity records were lower or higher than the deviations.

**
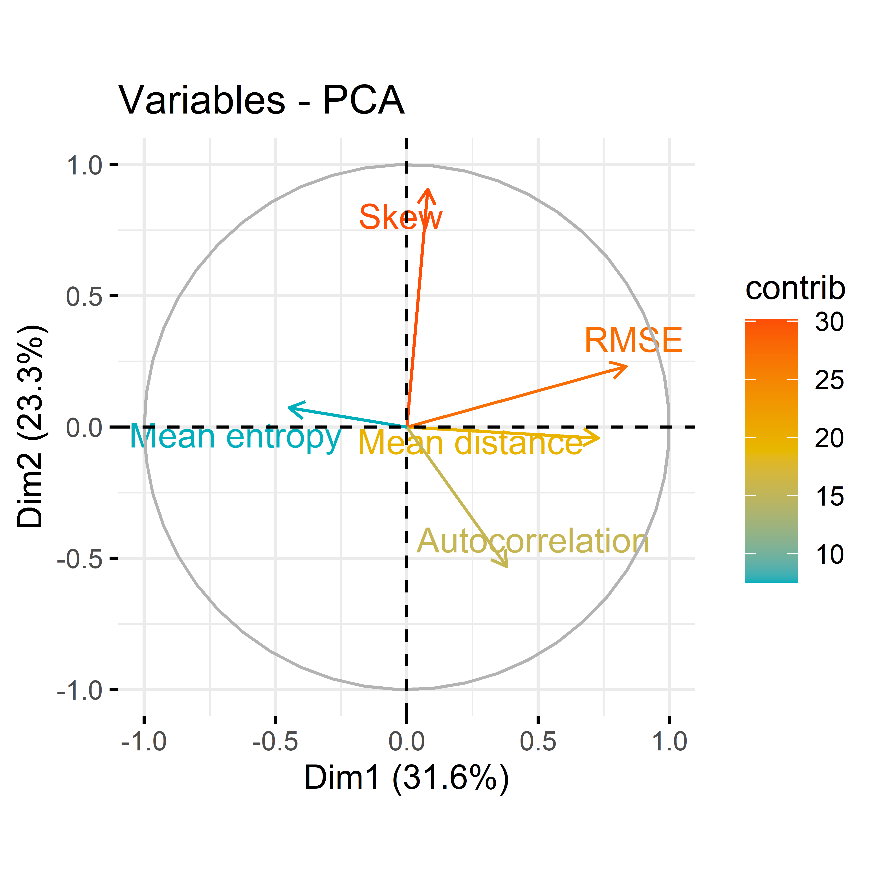
**

Figure S3.1: Overview of the contributions of the activity descriptors to the first two dimensions of the PCA.

**Reference**

Kassambara, A., and F. Mundt. 2020. factoextra: extract and visualize the results of multivariate data analyses. R package version 1.0.7. Available online: https://CRAN.R-project.org/package=factoextra
